# Continuity in palliative care – analysis of intersectoral palliative care based on routine data of a statutory health insurance

**DOI:** 10.1186/s12904-021-00751-0

**Published:** 2021-04-13

**Authors:** Laura Rehner, Kilson Moon, Wolfgang Hoffmann, Neeltje van den Berg

**Affiliations:** Department of Epidemiology and Community Health, Institute for Community Medicine, University Medicine Greifswald, Ellernholzstr. 1-2, 17489 Greifswald, Germany

**Keywords:** Palliative care, Hospice, Continuity of patients care, Intersectoral palliative care, Rural, Urban, Claims data

## Abstract

**Background:**

The goal of palliative care is to prevent and alleviate a suffering of incurable ill patients. A continuous intersectoral palliative care is important. The aim of this study is to analyse the continuity of palliative care, particularly the time gaps between hospital discharge and subsequent palliative care as well as the timing of the last palliative care before the patient’s death.

**Methods:**

The analysis was based on claims data from a large statutory health insurance. Patients who received their first palliative care in 2015 were included. The course of palliative care was followed for 12 months. Time intervals between discharge from hospital and first subsequent palliative care as well as between last palliative care and death were analysed. The continuity in palliative care was defined as an interval of less than 14 days between palliative care. Data were analysed using descriptive statistics and Chi-Square.

**Results:**

In 2015, 4177 patients with first palliative care were identified in the catchment area of the statutory health insurance. After general inpatient palliative care, 415 patients were transferred to subsequent palliative care, of these 67.7% (*n* = 281) received subsequent care within 14 days. After a stay in a palliative care ward, 124 patients received subsequent palliative care, of these 75.0% (*n* = 93) within 14 days. Altogether, 147 discharges did not receive subsequent palliative care. During the 12-months follow-up period, 2866 (68.7%) patients died, of these 78.7% (*n* = 2256) received palliative care within the last 2 weeks of life. Of these, 1223 patients received general ambulatory palliative care, 631 patients received specialised ambulatory palliative care, 313 patients received their last palliative care at a hospital and 89 patients received it in a hospice.

**Conclusions:**

The majority of the palliative care patients received continuous palliative care. However, there are some patients who did not receive continuous palliative care. After inpatient palliative care, each patient should receive a discharge management for a continuation of palliative care. Readmissions of patients after discharge from inpatients palliative care can be an indication for a lack of support in the ambulatory health care setting and for an insufficient discharge management. Palliative care training and possibilities for palliative care consultations by specialists should strengthen the GPs in palliative care.

**Supplementary Information:**

The online version contains supplementary material available at 10.1186/s12904-021-00751-0.

## Background

Palliative care is an approach to improve the quality of life of patients and their relatives, who are confronted with a life-threatening illness. The aim of palliative care is to prevent and to alleviate a suffering of incurable ill patients and their relatives through physical, psychosocial and spiritual support [1, 2]. Each patient should receive access to palliative care whenever it is needed regardless of diagnosis, age, place of residence and economic aspects [3, 4].

In the German health care system, palliative care is available on different levels and provided by different healthcare providers. General practitioners (GP) and ambulatory nursing services provide general ambulatory palliative care. Multi-professional specialised palliative care teams provide specialised ambulatory palliative care [5, 6]. General inpatient palliative care is provided by physicians and nurses without training in palliative care on general wards supervised by a physician specialised in palliative medicine. Specialised inpatient palliative care is provided by multi-professional teams trained in palliative care on palliative care wards. Patients in advanced stages of their disease, who cannot be cared for in their own homes, also have the possibility to move into a hospice. In these facilities, the patients receive specialised palliative care by trained nurses and ambulatory palliative care by GPs or from physicians of a specialised ambulatory palliative care team [7]. For patients, continuous, uninterrupted and intersectoral palliative care with no gaps of more than 14 days is particularly important. This is the only way to ensure adequate symptom management, prevent a patient’s suffering and establish a relationship of trust [7, 8].

Mecklenburg-Western Pomerania, a federal state in northeast Germany, is characterised by an increasing number of elderly people and a low population density with on average 69 persons per square kilometre [9, 10]. Due to the low population density and low number of health care providers in rural areas of the federal state, the distances between health care providers and patients are often large [11]. A continuous, intersectoral and uninterrupted supply of palliative care with no breaks of more than 14 days is a challenging task that can only be achieved through collaborative, multi-professional, ambulatory and inpatient care covering the whole range from primary to palliative care [12, 13]. Discharge from the hospital is a critical phase for palliative care patients. Subsequent ambulatory palliative care depends on local factors like the presence of relatives as care givers, the social situation as well as the availability of trained palliative care professionals [14]. Little is known about the time intervals over which palliative care patients are medically treated, especially when changing from the inpatient to the ambulatory setting and back, and before death.

The aim of this study is to analyse the continuity of palliative care, particularly the time gaps between hospital discharge, hospital readmissions and subsequent palliative care as well as the timing of the last palliative care before the patient’s death in the federal state of Mecklenburg-Western Pomerania in Germany. Due to the geographical characteristics of Mecklenburg-Western Pomerania, differences of the utilisation of palliative care between urban and rural areas were also of interest.

## Methods

### Statutory health insurance AOK-Nordost

In Germany, having a health insurance is compulsory for all citizens either through a statutory or a private health insurance. About 88% of the population have a statutory health insurance and a further 11% have a private health insurance [15]. The analysis was based on claims data from the AOK-Nordost, which is a large statutory health insurance provider in the federal states of Berlin, Brandenburg and Mecklenburg-Western Pomerania, Germany. In the federal state of Mecklenburg-Western Pomerania, the AOK-Nordost insured 431,474 people in 2015, which represents 27% of the population. In this region, the AOK-Nordost insures elderly people to a large part. Data from the years 2015 and 2016 were used for the analysis.

### Claims data

The claims data included pseudonymised information on demographic parameters (age, sex, date of death, and place of residence), ambulatory care (treatment codes, treatment date, diagnosis codes, in-home nursing care service and treatment of specialised ambulatory palliative care), inpatient hospice (admission and discharge dates) and hospital care (treatment dates, admission and discharge dates, main diagnoses, and operations and procedures codes). However, reimbursement data do not provide information on the provision of palliative care by ambulatory nursing services because there is no specific reimbursement code for palliative care services. Furthermore, the claims data did not include information on volunteer palliative care services because only reimbursed services are included in the data.

### Definition of palliative care

To determine general ambulatory palliative care, the codes for palliative care services from the reimbursement catalogue of the statutory health insurances for ambulatory care (EBM) were applied. To identify specialised ambulatory palliative care, data from special contracts with palliative care teams including the kind of treatment and dates of the home visits were used. In-hospital palliative care was identified on the basis of codes for operations and procedures that revealed general inpatient palliative care and treatment in a palliative care ward.

### Definition of continuity of palliative care

The German S3-guideline on palliative care for oncological patients defines continuous care as the standard for palliative care, but does not specify a time period [8]. Reimbursement data was used to analyse the continuity of care. In this data, there may be differences of a few days between the reimbursement date and the data of actual palliative care in some cases. This may cause bias when using small periods of time. Because of this potential bias, continuity of palliative care was defined as an interval of less than 14-days between different kinds of palliative care services for the present analysis.

### Patient selection

**Fig. 1:**
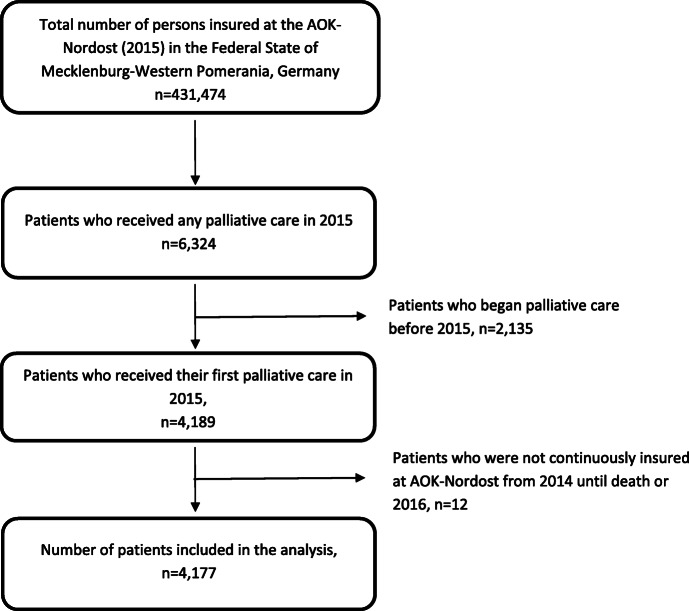
Flow-chart of patient selection

Patients who received palliative care for the first time in 2015 were included in the analysis. In addition, patients had to be continuously insured by the statutory health insurance (AOK-Nordost) from 2014 until death or for at least 12 months after starting palliative care. This resulted in a final study population of 4177 palliative care patients. Details about the sample collection are provided in Fig. 1.

### Diagnoses

The patient’s diagnoses were analysed by using the international Statistical Classification of Diseases (ICD) codes (10th revision, German modification). The diagnoses of the study population were the hospital diagnoses (principal and secondary diagnoses), and ambulatory diagnoses were used if they were present in at least two quarters of a single year (M2Q criterion).

### Urban/rural regions

For the study, the patients were divided into two groups based on their postal code of residence: those from rural areas, and those from urban areas. The regional centres in the federal state of Mecklenburg-Western Pomerania are Rostock, Schwerin, Neubrandenburg and Stralsund/Greifswald as a joint regional centre. These regional centres all have more than 50,000 inhabitants and are considered as urban areas. Other areas are considered as rural areas.

### Statistical analyses

All statistical analyses were performed using SAS® (Version 9.4; SAS Institute, Cary, NC, USA). Descriptive results are expressed as percentages, absolute numbers and medians with interquartile ranges (IQR). A Chi-Square test was used to compare the categorical variables. A *p*-value of less than 0.05 was considered statistically significant. The data analysis followed the guidelines and recommendations for ensuring “Good Epidemiological Practice” [16] and the guidelines of “Good Practice Secondary Data Analysis” [17].

### Ethics

The present study is based on a retrospective analysis of pseudonymised health insurance claims data and therefore no formal ethics committee approval was needed [17]. The data provider AOK-Nordost provided pseudonymised data, the authors did not have access to any identifiable data. Permission to transfer social data for research was obtained by AOK-Nordost from the responsible supervisory authority (the Ministry of Labour, Social Affairs, Health, Women and Family of the federal state of Brandenburg (Germany)) in accordance with §73 of social code X (SGB X).

## Results

### Characteristics and total number of palliative care patients

Table 1 shows the characteristics of the palliative care patients in urban and rural areas in Mecklenburg-Western Pomerania in 2015 and 2016, which were followed for 12 months. Four thousand one hundred seventy-seven palliative care patients were identified. The median age of the palliative care patients was 81.0 (IQR 74.0–87.0) years, and 54.6% (*n* = 2280) were female. Of the palliative care patients, 59.3% (*n* = 2477) had oncological diagnoses. 68.6% (*n* = 2866) patients died during the 12-month observation period. The palliative care of the deceased patients lasted a median of 29.0 days (IQR 8.0–83.0). Overall, 85.7% (*n* = 3579) of the palliative care patients received general and 21.6% (*n* = 904) received specialised ambulatory palliative care at least once. General inpatient palliative care was received by 14.6% (*n* = 611) of the patients, and 5.1% (*n* = 214) were treated on a palliative care ward in hospital. In total 2.7% (*n* = 112) of the patients were cared for in a hospice (Table 1).

**Table 1:** Characteristics of patients with palliative care and total number patients receiving inpatient and ambulatory palliative care in 2015/16, 12-month follow-up

	Urban		Rural		Total	
	n	%	n	%	n	%
**Total number of palliative patients**	1004	100.0	3173	100.0	4177	100.0
**Age (y)**						
Median (IQR)	82.0 (74.0, 88.0)		81.0 (73.0, 87.0)		81.0 (74.0, 87.0)	
**Sex**						
Male	416	41.4	1481	46.7	1897	45.4
Female	588	58.6	1692	53.3	2280	54.6
**Deceased (until 12 months treatment)**						
	743	74.0	2123	66.9	2866	68.6
**Ambulatory and stationary diagnoses**	974	97.0	3088	94.8	4062	97.3
Oncological diagnoses	589	58.7	1888	59.5	2477	59.3
**General ambulatory palliative care**						
Number of palliative care services	2761		11,815		14,576	
Number of patients	785	78.2*	2794	88.1*	3579	85.7
**Specialised ambulatory palliative care**						
Number of patients	261	26.0*	643	20.3*	904	21.6
Median (IQR) treatment days	14.0 (6.0, 41.0)		19.0 (7.0, 53.0)		17.0 (7.0, 49.0)	
**General inpatient palliative care**						
Number of palliative cases	205		552		757	
Number of patients	174	17.3*	437	13.8*	611	14.6
Median (IQR) length of stay (d)	13.0 (8.0, 21.0)		14.0 (10.0, 22.0)		14.0 (9.0, 22.0)	
Palliative care ward						
Number of palliative cases	96		156		252	
Number of patients	82	8.2*	132	4.2*	214	5.1
Median (IQR) length of stay (d)	16.5 (9.5, 27.0)		15.0 (7.5, 24.0)		13.0 (8.0, 23.0)	
**Hospice**						
Number of patients	46	4.6*	66	2.1*	112	2.7
Median (IQR) length of stay (d)	27.0 (18.0, 59.0)		40.5 (26.0, 81.0)		37.5 (20.5, 70.0)	

*IQR* interquartile range (lower quartile, upper quartile)

*p < 0.001 with Chi-Square test among urban and rural areas

### Palliative care in rural and urban areas

In urban areas lived 24% (*n* = 1004) of the identified patients with palliative care and 76% (*n* = 3173) lived in rural areas.

More patients in rural areas received general outpatient palliative care (88.1% vs 78.2%) than in urban areas (*p* < 0.001). More patients in urban areas received specialised outpatient palliative care (26.0% vs 20.3%), general inpatient palliative care (17.3% vs 13.8%) and hospice care (4.6% vs 2.1%) than in rural areas (*p* < 0.001) (Table 1).

### Time interval between hospital discharge and consecutive palliative care

Table 2 shows the time interval between the hospital discharge and the start of consecutive palliative care. The table presents the number of discharges rather than patients since some patients were discharged several times from the hospital, and all discharges were included. In 415 discharges, subsequent palliative care after general inpatient palliative care was documented. After the discharge from general inpatient palliative care, general ambulatory palliative care was provided in 21.9% (*n* = 91) of the discharges and specialised ambulatory palliative care in 34.5% (*n* = 143) within 14 days. Most of the patients who received subsequent palliative care within 14 days (discharge form general inpatient palliative care) had oncological diagnoses (51.3%; *n* = 213) (Additional file 1) Readmissions in the hospital after general inpatient palliative care, whether it was planned or unplanned is unknown, happened in 21.7% (*n* = 90) of the discharges, 6.0% (*n* = 25) received general palliative care and 4 were treated on a palliative care ward within 14 days. Within 14 days after the hospital discharge from general inpatient palliative care similar cases from urban areas (69.3%) received subsequent palliative care than cases from rural areas (67.2%) (Table 2).

**Table 2:** Time interval between discharge from the hospital (general palliative care and palliative care ward) and subsequent palliative care

	≤ 14 days		15–28 day		> 28 days	
	n	%	n	%	n	%
From general inpatient palliative care to:						
Total number of discharges, *n* = 415*	281	67.7	46	11.1	88	21.2
General ambulatory palliative care, *n* = 130	91	21.9	14	3.4	25	6.0
Specialised ambulatory palliative care, *n* = 176	143	34.5	14	3.4	19	4.6
Stationary readmission, *n* = 90	29	7.0	18	4.3	43	10.4
- *General inpatient palliative care, n = 76*	25	6.0	17	4.1	34	8.2
- *Palliative care ward, n = 14*	4	1.0	1	0.2	9	2.2
Hospice, *n* = 19	18	4.3	0	0.0	1	0.2
From palliative care ward to:						
Total number of discharges, *n* = 124**	93	75.0	13	10.5	18	14.5
General ambulatory palliative care, *n* = 35	26	21.0	5	4.0	4	3.2
Specialised ambulatory palliative care, *n* = 62	55	44.4	1	0.8	6	4.8
Stationary readmission, *n* = 23	10	8.1	5	4.0	8	6.5
- *General inpatient palliative care, n = 10*	*3*	*2.4*	*3*	*2.4*	*4*	*3.2*
- *Palliative care ward, n = 13*	*7*	*5.6*	*2*	*1.6*	*4*	*3.2*
Hospice, *n* = 4	2	1.6	2	1.6	0	0.0

* ≤ 14 days: urban, 69.3%; rural, 67.2% (*p* > 0.05 with Chi-Square test among urban and rural areas)

** ≤ 14 days: urban, 75.6%; rural, 74.7% (*p* > 0.05 with Chi-Square test among urban and rural areas)

In 124 discharges, subsequent palliative care after treatment on a palliative care ward was utilised. Most of the patients with subsequent palliative care (discharge from palliative care ward) within 14 days had oncological diagnoses (59.7%; *n* = 74) (Additional file 1). After discharge from a palliative care ward, subsequent general ambulatory palliative care was provided in 21.0% (*n* = 26) of the discharges and specialised ambulatory palliative care in 44.4% (*n* = 55) of the discharges within 14 days. Rehospitalisation after a stay on a palliative care ward happened in 18.5% (*n* = 23) of the discharges, 10 cases received inpatient palliative care again within 14 days. There is no difference in the proportion of cases in urban (75.6%) and rural areas (74.7%) as both groups received subsequent palliative care within 14 days after hospital discharge (Table 2).

### No further palliative care after inpatient palliative care

In total, 539 discharges after inpatient palliative care were documented. For 114 discharges, no subsequent palliative care was documented (Table 3). Of these, 107 (93.9%) received subsequent general care by ambulatory nursing services (*n* = 70, 61.4%), in the hospital (*n* = 50, 43.9%) or by the GP (*n* = 101, 88.6%). Three of the seven cases with no other medical care after hospital discharge died within less than 7 days after discharge from general inpatient palliative care (Table 3).

**Table 3:** No subsequent palliative care after last inpatient palliative care

	General inpatient palliative care		Palliative care ward	
	n	%	n	%
After leaving hospital no further palliative care	114	100.0	33	100.0
Receiving *no* other treatment	7^a^	6.1	4^b^	12.1
Receiving non-palliative treatment inpatient or ambulatory sector	107	93.9	29	87.9
- *Ambulatory nursing services*	70	61.4	25	75.8
- *In hospital*	50	43.9	13	39.4
- *General practitioner*	101	88.6	28	84.8

^a^Time interval between hospital discharge and death: < 7 days: *n* = 3: alive, *n* = 4

^b^Time interval between hospital discharge and death: < 7 days: *n* = 2: alive, *n* = 2

After treatment on a palliative care ward, 33 discharges received no subsequent palliative care. Of these, 29 (87.9%) received subsequent general care by ambulatory nursing services (*n* = 25, 75.8%), in hospital (*n* = 13, 39.4%) or by the GP (*n* = 28, 84.8%) (Table 3). Two of the four cases with no subsequent medical care died within less than 7 days after discharge from a palliative care ward.

### Time interval between last palliative care and death

Table 4 shows the time interval between the last provision of palliative care and the patient’s death. Altogether, 68.6% (*n* = 2866) of the palliative care patients died within the 12-months follow-up period. Of these patients, 58.8% (*n* = 1713) received general ambulatory palliative care and 22.9% (*n* = 656) specialised ambulatory palliative care at least once before dying. General ambulatory palliative care received 71.4% (*n* = 1223) of the patients within the last 14 days of their life. Specialised ambulatory palliative care received 96.2% (*n* = 631) of the patients within the last 14 days of their life (Table 4). Before dying 14.2% (*n* = 408) of the patients received their last palliative care at a hospital. Of these 72.8% (*n* = 297) received general inpatient palliative care, and 27.2% (*n* = 111) were on a palliative care ward. General inpatient palliative care received 73.1% (*n* = 217) of the patients within 14 days before death. Care at a palliative care ward received 86.5% (*n* = 96) of the patients within 14 days before death.

**Table 4:** Time interval between last palliative care and death, 12-months follow-up

	≤ 14 days		15–28 days		> 28 days	
	n	%	n	%	n	%
Dead before 12-months follow-up, ***n*** = 2866*	2256	78.7	268	9.4	342	11.9
General ambulatory palliative care, ***n*** **= 1713**	1223	71.4	201	11.7	289	16.9
Specialised ambulatory palliative care**, ***n*** **= 656**	631	96.2	10	1.5	15	2.3
Hospital, ***n*** **= 408**	313	76.7	57	14.0	38	9.3
- *General inpatient palliative care,* ***n = 297***	*217*	*73.1*	*48*	*16.2*	*32*	*10.8*
- *Palliative care ward,* ***n = 111***	*96*	*86.5*	*9*	*8.1*	*6*	*5.4*
Hospice***, ***n*** **= 89**	89	100.0	0	0.0	0	0.0

* ≤ 14 days: urban, 78.5%; rural, 78.8% (*p* > 0.05 with Chi-Square test among urban and rural areas)

** ≤ 7 days: *n* = 622 (0 day: *n* = 509)

*** ≤ 7 days: *n* = 87 (0 day: *n* = 75)

In terms of the last palliative care within 14 days before death, it made no difference whether the patients lived in urban (78.5%) or rural (78.8%) areas (Table 4).

## Discussion

The majority of discharges from inpatient palliative care received subsequent palliative care within 14 days. Most of the patients with subsequent palliative care were patients with oncological diagnoses (Additional file 1). After inpatient palliative care (general or palliative care ward), specialised ambulatory palliative care was provided more often within 14 days than general ambulatory palliative care. In some discharges, no subsequent palliative care was provided after general inpatient palliative care, however treatment in the setting of primary care was provided. Only a small number of discharges from a palliative care ward did not receive any subsequent palliative care. Some inpatient palliative care cases did not receive subsequent ambulatory palliative care and were treated again in hospital within 28 days after discharge. The results show a difference in the number of treated patients between urban and rural regions but no differences with respect to the continuity of palliative care. Readmission of patients after discharge from inpatients palliative care can be an indication of insufficient discharge management and a lack of support in the ambulatory health care setting [18]. Other possible risk factors for hospital readmissions within 30 days include neoplasms, prescribed opiates, comorbidities and an increased number of hospitalisations in the past [19].

The majority of patients died during the 12-month observation period. Specialised ambulatory palliative care was provided more frequently than general ambulatory palliative care within the last 14 days of life. In terms of the time interval between subsequent care or death, there was no difference whether the patients lived in rural or urban areas.

In Germany, the treatment on a palliative care ward includes the organisation of subsequent palliative care after discharge. In contrast, this is not required after inpatient palliative care at a general ward [20]. This may explain the results of the study that more discharges from a palliative care ward received subsequent specialised palliative care within 14 days, compared to general inpatient palliative care. Scott et al. [18] describe that inpatient palliative care consultations can support the discharge from hospital into ambulatory palliative care by discharge planning and advanced care planning. Inpatient palliative care consultation is associated with a lower number of readmissions compared to care as usual without palliative care. It is also associated with a higher number of admissions to hospices compared to those with inpatient care as usual [18]. However, it should be noted that the majority of palliative care patients were oncological patients. Oncological diseases are mentioned as a risk factor for hospital readmissions within 30 days, and there are also indications that these readmissions may not be prevented [19, 21]. To prevent unnecessary readmissions to hospitals and many changes between the types of care as well as to improve the patients’ and their relatives’ wellbeing, the discharge management should be well organised. A close cooperation between providers of general and specialised palliative care is necessary as well as advanced care planning [21, 22]. This kind of organisation should also be available for patients with general inpatient palliative care. Nevertheless, the organisation of ambulatory palliative care depends on existing ambulatory health care providers [14]. The results show that some patients did not receive subsequent palliative care. They might be cared for by ambulatory nursing services, by GPs or in hospitals without palliative care. This interruption of palliative care could have several reasons. One reason might be that the specialised ambulatory palliative care teams and hospices were unable to care for the patient or that the GP did not recognise the need for palliative care. Another reason could be that patients refused palliative care. On the basis of claims data, it cannot be determined whether a patient’s needs-based care has actually been provided. Patients who receive ambulatory palliative care are more likely to die at home. GPs report that during the last days of the patient’s life, the intensity of their care is usually growing [23]. This cannot be fully transferred to the results of our analysis. The majority of patients received general ambulatory palliative care by GPs. The duration between the last general ambulatory palliative care and death is longer than in specialised ambulatory palliative care. More patients received specialised ambulatory palliative care than general ambulatory palliative care within the last 14 days of life. In this case, it makes no difference whether the patient lived in rural or urban regions.

The high number of patients with general ambulatory palliative care in the results emphasise the role of GPs in palliative care. GPs have a key role in identifying the need for palliative care and deciding which type of care suits the patient best. GPs see palliative care as an important, albeit small part of their work. They define their part in palliative care as coordinators and referrers to specialised palliative care, but still some GPs may have little knowledge about palliative care and the structures of specialised palliative care [23, 24]. However, some GPs are concerned about losing their care mandate to palliative care specialists [24]. In contrast, the high bureaucratic demands and the time-consuming home visits, lack of skills and confidence make it difficult to provide proper ambulatory palliative care [24, 25]. However, the number of patients with general ambulatory palliative care provided by the GPs as well as the number of patients, who are still alive at the end of the 12-month follow up is high. The results may show an indication for overprovision or even misprovision of general ambulatory palliative care and an underprovision of specialised ambulatory palliative care, especially in rural areas. Specialised ambulatory palliative care teams are more likely to be located in urban areas [11]. There is a possibility that if a specialised ambulatory palliative care team is nearby, patients are more likely to be referred to this kind of care [26]. Palliative care providers in Mecklenburg-Western Pomerania are geographically far away from the patients and therefore time-consuming home visits are common. In this case, specialised ambulatory palliative care teams have to travel up to 30 km to care for patients in rural areas [11]. The results show that subsequent specialised ambulatory palliative care after a hospital stay within 14 days is more often provided in urban areas. When organising subsequent palliative care, the local healthcare structures must be taken into account [22].

Due to the aging population, the number of palliative care patients who will have to be cared for by the existing health and palliative care providers will increase [27]. In an ambulatory setting, continuous care and monitoring cannot always be provided due to the lack of resources [28]. Innovative solutions are needed to meet the need for ambulatory palliative care, especially in rural areas. The applicability and feasibility of telemedicine and mobile health technologies for palliative care patients and their caregivers should be evaluated. The claims data do not give any information about palliative care provided by ambulatory nursing services. Like GPs, they are likely to make an important contribution to general ambulatory palliative care, but little is known about the kind and level of palliative care provided by nursing services. More research about this topic is needed.

### Strengths and limitations

In total 27% of the population in Mecklenburg-Western Pomerania are insured with the statutory health insurance company AOK-Nordost, therefore, the results may not be representative for the entire population. A limitation of claims data is that these data were collected for reimbursement purposes and that they may not represent the healthcare situation completely or accurately. A strength of this study is that a large part of the insured people are elderly people, as most of the palliative patients belong to the older population. Another strength is the availability of both inpatient and ambulatory data. The data allow to study the course of the patients through the healthcare system.

### Conclusions

The majority of the palliative care patients in Mecklenburg-Western Pomerania receive continuous palliative care. For a continuation of palliative care and a smooth transition to a patient’s home after a hospital stay, each patient with inpatient palliative care should receive a discharge management, regardless of the severity of his or her disease. More research should be done about the needs of patients with only general ambulatory palliative care. Innovative solutions for the provision of specialised palliative care in rural regions should be developed. Palliative care training and possibilities for palliative care consultations by specialists should strengthen the GPs’ gatekeeper role in palliative care.

## Supplementary Information

**Additional file 1: Appendix Table A.** Discharge diagnoses from general inpatient palliative care and palliative care ward differentiated by time interval between these discharges and first further palliative care.

## Data Availability

The data that support the findings of this study are available at AOK-Nordost but restrictions apply to the availability of these data, which were used under license for the current study, and so are not publicly available. Data are however available from the authors upon reasonable request and with permission of AOK-Nordost.
