# Supplementary material for: Continuity in palliative care – analysis of intersectoral palliative care based on routine data of a statutory health insurance
Source: BMC Palliat Care. 2021 Apr 13;20:59. doi: 10.1186/s12904-021-00751-0 (PMC8045326; doi:10.1186/s12904-021-00751-0)
Supplement: Supplementary file 1 — Additional file 1: Appendix Table A. Discharge diagnoses from general inpatient palliative care and palliative care ward differentiated by time interval between these discharges and first further palliative care. [file 12904_2021_751_MOESM1_ESM.docx]

**APPENDIX**

**Table A:** Discharge diagnoses from general inpatient palliative care and palliative care ward differentiated by time interval between these discharges and

first further palliative care

|  | General inpatient palliative care | | | | | |  | Palliative care ward | | | | | | |
| --- | --- | --- | --- | --- | --- | --- | --- | --- | --- | --- | --- | --- | --- | --- |
|  | ≤ 14 days | | 15-28 day | | > 28 days | |  | ≤ 14 days | | 15-28 day | | > 28 days | | |
| ICD-10 Chapter | n | % | n | % | n | % |  | n | % | n | % | n | % |  |
| Certain infectious and parasitic diseases | 6 | 2.1 | 5 | 10.9 | 3 | 3.4 |  | 6 | 6.5 | 2 | 15.4 | 1 | 5.6 |  |
| Neoplasms | 213 | 75.8 | 32 | 69.6 | 54 | 61.4 |  | 74 | 79.6 | 8 | 61.5 | 11 | 61.1 |  |
| Diseases of the blood and blood-forming organs and certain disorders involving the immune mechanism | 1 | 0.4 | 1 | 2.2 | 0 | 0.0 |  | 0 | 0.0 | 1 | 7.7 | 1 | 5.6 |  |
| Endocrine, nutritional and metabolic diseases | 6 | 2.1 | 2 | 4.3 | 3 | 3.4 |  | 0 | 0.0 | 0 | 0.0 | 1 | 5.6 |  |
| Mental and behavioural disorders | 1 | 0.4 | 0 | 0.0 | 0 | 0.0 |  | 0 | 0.0 | 0 | 0.0 | 0 | 0.0 |  |
| Diseases of the nervous system | 1 | 0.4 | 1 | 2.2 | 1 | 1.1 |  | 0 | 0.0 | 0 | 0.0 | 0 | 0.0 |  |
| Diseases of the circulatory system | 16 | 5.7 | 0 | 0.0 | 7 | 8.0 |  | 2 | 2.2 | 1 | 7.7 | 3 | 16.7 |  |
| Diseases of the respiratory system | 13 | 4.6 | 1 | 2.2 | 7 | 8.0 |  | 4 | 4.3 | 1 | 7.7 | 1 | 5.6 |  |
| Diseases of the digestive system | 17 | 6.0 | 4 | 8.7 | 9 | 10.2 |  | 1 | 1.1 | 0 | 0.0 | 0 | 0.0 |  |
| Diseases of the skin and subcutaneous tissue | 1 | 0.4 | 0 | 0.0 | 0 | 0.0 |  | 1 | 1.1 | 0 | 0.0 | 0 | 0.0 |  |
| Diseases of the musculoskeletal system and connective tissue | 0 | 0.0 | 0 | 0.0 | 1 | 1.1 |  | 0 | 0.0 | 0 | 0.0 | 0 | 0.0 |  |
| Diseases of the genitourinary system | 3 | 1.1 | 0 | 0.0 | 1 | 1.1 |  | 2 | 2.2 | 0 | 0.0 | 0 | 0.0 |  |
| Other | 3 | 1.1 | 0 | 0.0 | 2 | 2.3 |  | 3 | 3.2 | 0 | 0.0 | 0 | 0.0 |  |
| Total | 281 | 100 | 46 | 100 | 88 | 100 |  | 93 | 100 | 13 | 100 | 18 | 100 |  |
